# Gaussian Accelerated Molecular Dynamics Simulations Combined with NRIMD to Explore the Mechanism of Substrate Selectivity of Cid1 Polymerase for Different Nucleoside Triphosphates

**DOI:** 10.3390/ijms26199325

**Published:** 2025-09-24

**Authors:** Hanwen Liu, Xue Zhou, Haohao Wang, Fuyan Cao, Weiwei Han

**Affiliations:** Key Laboratory for Molecular Enzymology and Engineering of Ministry of Education, School of Life Sciences, Jilin University, Changchun 130012, China; u5811963abc@126.com (H.L.); zhouxue24@mails.jlu.edu.cn (X.Z.); haohao24@mails.jlu.edu.cn (H.W.); caofy22@mails.jlu.edu.cn (F.C.)

**Keywords:** GaMD, molecular docking, substrate specificity, Cid1 polymerase, NRIMD

## Abstract

Cid1 protein is a crucial component in the RNA interference pathway and abnormal nuclear RNA turnover processes, primarily responsible for adding uridine to the 3′ end of RNA. Cid1 exhibits selective polymerization of UTP over other nucleoside triphosphates. To explore the mechanism of this selectivity, five systems: free-Cid1, Cid1-ATP, Cid1-UTP, Cid1-CTP, and Cid1-GTP with 500 ns Gaussian accelerated molecular dynamics (GaMD) simulations were performed to investigate conformational changes and binding affinities between substrates and Cid1. The results showed that UTP formed stronger and more numerous non-covalent interactions with Cid1 compared to the other three substrates. The Molecular Mechanics Poisson-Boltzmann Surface Area (MM-PBSA) binding energy analysis revealed a substrate preference for Cid1 polymerase in the order of UTP, followed by ATP, CTP, and GTP. These findings provide theoretical insights into the substrate selectivity mechanism of Cid1 and provide theoretical clues for the design and modification of Cid1 polymerase.

## 1. Introduction

RNA polymerase (RNAP) is a multisubunit protein complex that utilizes four ribonucleoside triphosphates (NTPs: ATP, GTP, CTP, and UTP) as substrates, DNA as a template, and divalent metal ions such as Mg^2+^, serve as essential cofactors. It polymerizes nucleotides via phosphodiester bonds to convert DNA-encoded genetic information into RNA [1,2]. Eukaryotic RNA polymerases are categorized into three types: Pol I, Pol II, and Pol III, which exhibit significant differences in subunit composition, regulatory elements, and auxiliary factors, while collaborating with diverse cofactors to achieve precise transcription of their specific RNA products [3,4,5,6,7,8,9,10,11,12,13]. Template-independent RNA polymerases constitute a special class of RNA polymerases capable of directly synthesizing RNA molecules in the absence of an RNA template, exhibiting greater flexibility and adaptability. Moreover, due to their ability to synthesize RNA without requiring the prior design of specific template sequences, template-independent RNA polymerases hold significant value in biological research and applications.

As a template-independent RNA polymerase, Cid1 protein belongs to the nucleotidyltransferase superfamily [14]. Cid1 polymerase is the primary enzyme responsible for mRNA uridylation, which involves adding uridine residues to the 3′ end of RNA. This represents a non-canonical tail modification that is closely associated with gene expression regulation and RNA stability. Uridylation not only contributes to regulating RNA half-life and metabolic rates but also modulates mRNA degradation, transport, and translation [15,16,17,18]. Furthermore, it can regulate the production of histone mRNA, microRNA, siRNA, or U6 snRNA in the nucleus [19]. Therefore, the Cid1 protein plays a pivotal role in biological processes [20].

Cid1 plays an important role in biological processes such as the cell cycle, gene expression regulation, and post-transcriptional modifications. Previous studies indicated that Cid1 polymerase possesses poly(A) polymerase activity. However, In vitro experiments have demonstrated that the core function of Cid1 is that of a poly(U) polymerase (PUP), with its UTP preference predominating even under physiological nucleotide concentrations; Additionally, polyadenylated actin mRNA undergoes Cid1 polymerase-dependent uridylation during S-phase arrest [21]. Furthermore, By determining the structures of Cid1 in its apo state and in complex with four ribonucleoside triphosphates, Bradley M. Lunde et al. demonstrated that Cid1 exhibits a distinct substrate preference among the four NTPs: it shows the highest activity with UTP, followed by ATP, exhibits moderate activity with GTP, and negligible activity with CTP. Furthermore, the study identified H336 as a critical residue underlying this substrate preference [22]. Nevertheless, the molecular details underlying the kinetic mechanism of Cid1 substrate polymerization, particularly the specific mechanism of its substrate selectivity, remain unclear.

Molecular dynamics (MD) simulations have been frequently employed in research related to RNA polymerase. For instance, MD simulations have been used to analyze the interactions between RNA-dependent RNA polymerase (RdRp) and drugs, providing an effective approach for developing novel antiviral agents, such as those targeting SARS-CoV-2 [23,24,25,26,27]. However, the application of molecular dynamics simulations to investigate enzyme-substrate interactions, particularly those involving RNA polymerase and its substrates, remains relatively limited. Therefore, based on these experimental findings, this study employs MD simulation to conduct a more in-depth investigation into the underlying mechanisms.

This study designs the following five systems: Free-Cid1, Cid1-ATP, Cid1-UTP, Cid1-CTP, and Cid1-GTP. Gaussian accelerated molecular dynamics (GaMD) simulations are employed to systematically analyze the dynamic characteristics of Cid1. Furthermore, multiple approaches, including secondary structure analysis, Molecular Mechanics Poisson-Boltzmann Surface Area (MM-PBSA) calculations, NRIMD web server and Quantum Chemical Calculations, are combined to investigate the polymerization mechanism of Cid1. These findings provide theoretical insights into the substrate selectivity mechanism of Cid1 and provide theoretical clue for the design and modification of Cid1 polymerase.

## 2. Results and Discussion

### 2.1. Binding of Four Ligand Molecules to Cid1 Polymerase

**Figure 1:**
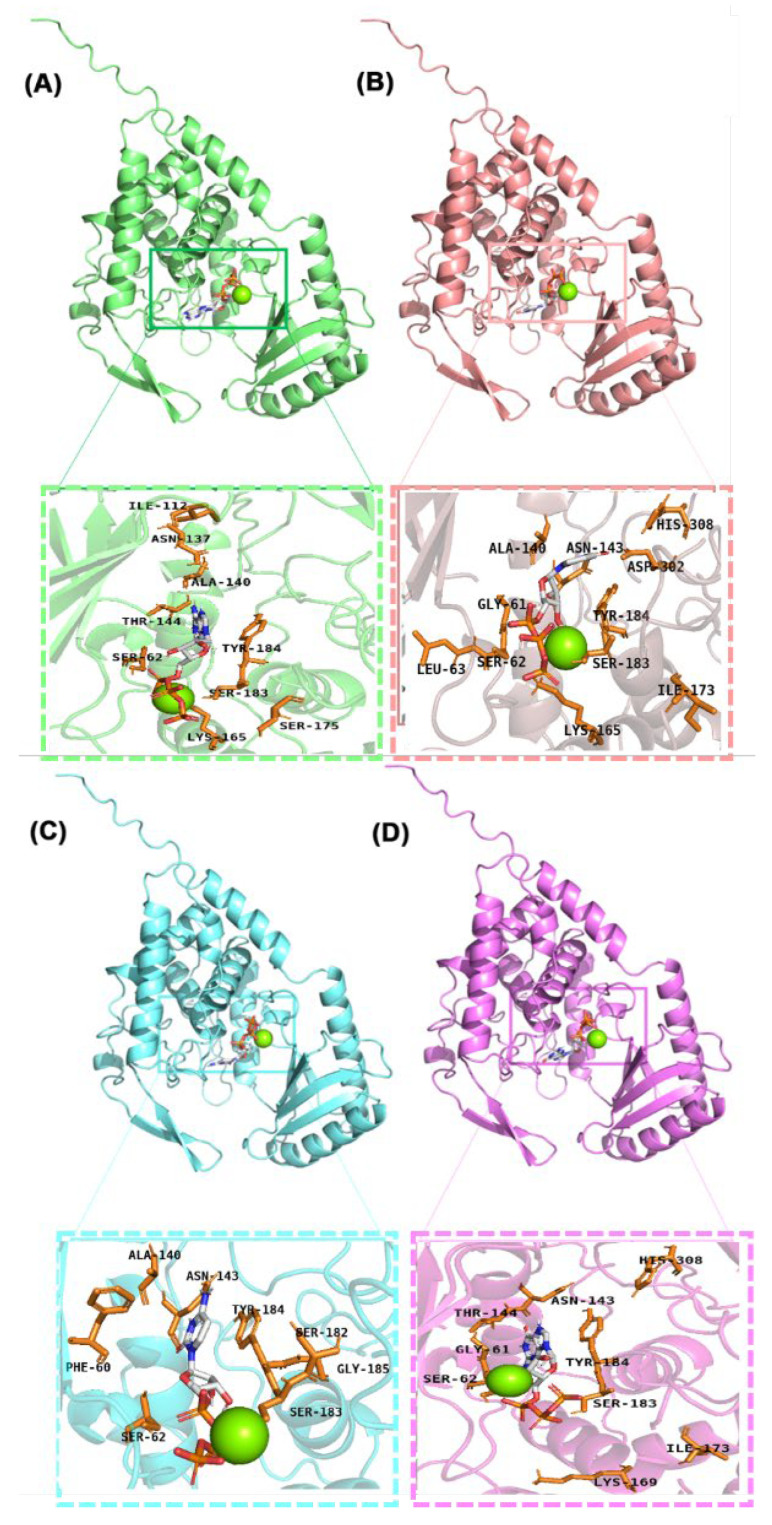
(**A**) ATP in Cid1 protein; (**B**) UTP in Cid1 protein; (**C**) CTP in Cid1 protein; (**D**) GTP in Cid1 protein.

Molecular docking is used to simulate the process of molecular recognition between multiple molecules through geometric complementarity and energetic compatibility [28]. By analyzing intermolecular forces and binding energies, molecular docking identifies optimal binding modes [29]. After obtaining the predicted structure of Cid1 polymerase using AlphaFold 3 [30], molecular docking analysis of the Cid1 protein with each of the four ligand molecules was performed. Figure 1A–D presents the molecular docking results for the four ligand molecules with the Cid1 protein, simultaneously identifying the amino acid residues involved in interactions surrounding the ligands. When ATP and UTP serve as substrates, the number of interacting residues between the ligand molecules and the protein is significantly greater than when CTP and GTP act as substrates. This observation suggests that the binding between the Cid1 protein and ATP/UTP may be tighter, potentially involving more extensive non-covalent interactions such as hydrogen bonds, van der Waals forces, and electrostatic interactions. Additionally, the docking conformations of the four NTP substrates all match the three main cavities of the enzyme’s binding pocket and consistently involve the key residues SER183, SER62, and TYR184, which play a central role in the binding between the substrates and the enzyme. In summary, these results confirm that UTP, ATP, CTP, and GTP effectively bind to the Cid1 pocket through interactions with conserved residues, providing reliable initial conformations for molecular dynamics simulations.

Further analysis revealed hydrogen bond formation between residue Ser62 in Cid1 and the ligands. To be specific, when UTP serves as substrate, two hydrogen bonds form between Ser62 and UTP, whereas only one hydrogen bond is observed with ATP, CTP, or GTP. This suggests residue Ser62 likely plays a critical role in UTP’s specific binding to Cid1 through the formation of a stable hydrogen bonding network. The stability of this network may directly influence Cid1’s binding affinity and catalytic efficiency toward different NTP substrates. Therefore we speculate that capacity of Ser62 to form substrate-specific hydrogen bonds may be a significant factor underlying UTP’s role as the preferred substrate for Cid1 polymerase.

### 2.2. Equilibration and Stability of Conventional MD Simulations

Prior to the GaMD production runs, all systems were subjected to a 50 ns conventional MD (cMD) simulation to achieve equilibration and to calculate parameters for the Gaussian acceleration. To validate that the systems reached a stable equilibrium state, we analyzed the root mean square deviation (RMSD) of the protein backbone and the radius of gyration (R_g_) during this cMD phase.

As shown in Supplementary Figure S1, the RMSD and R_g_ values for all five systems plateaued after approximately 30 ns (Figure S1A,C), indicating that the systems were well-equilibrated and no major unfolding or compaction events occurred. The sharp frequency distributions of both RMSD and R_g_ (Figure S1B,D) further confirm that each system predominantly sampled a stable conformational state. This confirmed that the systems had reached a stable state, providing a reliable foundation for the parameterization of the GaMD simulations and the subsequent production runs. The stability of these initial simulations ensures that the enhanced sampling in GaMD was applied to a representative, native-like conformational ensemble.

### 2.3. Protein Structural Stability Analysis

To assess the conformational stability of the complexes in the five simulated systems (Free-Cid1, Cid1-ATP, Cid1-UTP, Cid1-CTP, and Cid1-GTP), the study calculated the Root Mean Square Deviation (RMSD), Radius of Gyration (R_g_), and Solvent Accessible Surface Area (SASA) of the Cα atom trajectories during the three replicated 500 ns GaMD simulations. These metrics demonstrate that the MD simulations achieved sufficient stability at 310 K.

**Figure 2:**
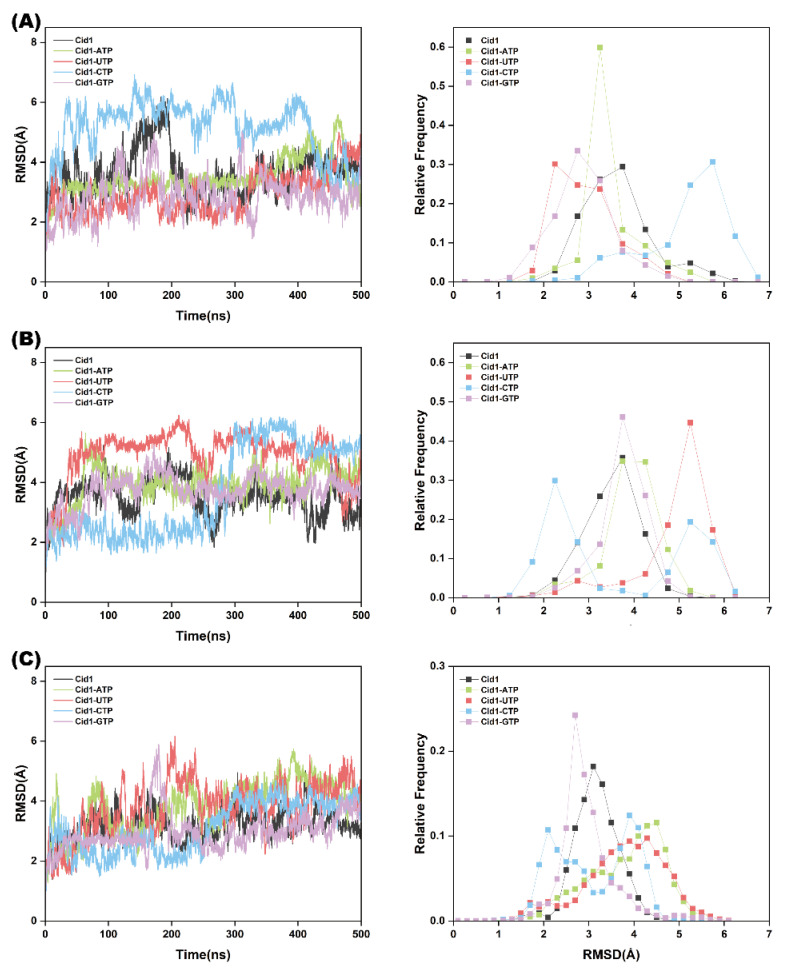
RMSD plots of the five systems (Free-Cid1, Cid1-ATP, Cid1-UTP, Cid1-CTP, and Cid1-GTP) from three independent simulation replicates. (**A**) Replicate 1; (**B**) Replicate 2; (**C**) Replicate 3.

RMSD is a crucial tool in MD simulations for evaluating system stability and convergence. RMSD effectively reflects conformational changes during the simulation, which is a powerful means for comprehending molecular behavior and dynamic characteristics, aiding researchers in understanding the extent of structural changes undergone by the molecule and whether it has converged to the expected conformation. As shown in Figure 2A, the RMSD of Cid1-UTP fluctuated around 2.86 Å. In contrast, the RMSD values for Cid1-ATP, Cid1-CTP, and Cid1-GTP fluctuated around 3.46 Å, 5.13 Å, and 2.92 Å, respectively. This indicates that, compared to the other three complexes, Cid1-UTP exhibited a lower overall RMSD and a more compact distribution, while the protein itself underwent no significant conformational changes. Figure 2B,C present the results from the second and third independent replicates, respectively. The relative consistency of the RMSD trends across all five systems is maintained in all replicates.

**Figure 3:**
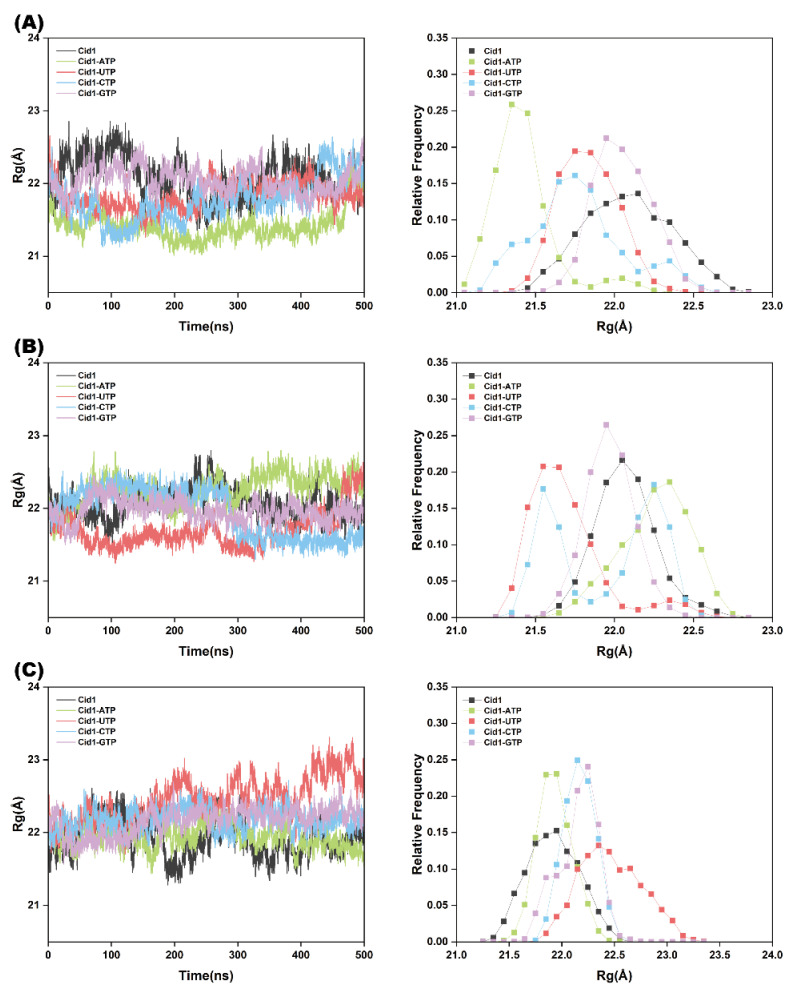
R_g_ plots of the five systems (Free-Cid1, Cid1-ATP, Cid1-UTP, Cid1-CTP, and Cid1-GTP) from three independent simulation replicates. (**A**) Replicate 1; (**B**) Replicate 2; (**C**) Replicate 3.

R_g_ is a parameter measuring the compactness of biomacromolecular structures. As shown in Figure 3A, the R_g_ values for the four complexes (Cid1-UTP, Cid1-ATP, Cid1-CTP, and Cid1-GTP) predominantly fluctuated around 21.77 Å, 21.55 Å, 21.83 Å, and 22.05 Å, respectively. Notably, both Cid1-ATP and Cid1-UTP complexes showed significantly smaller R_g_ fluctuations compared to the other complexes. This observation, together with their narrower R_g_ frequency distributions, suggests that these two complexes exhibit relatively compact conformations. Furthermore, an analysis of the R_g_ frequency distributions across all three independent simulations (Figure 3A–C) suggests a tendency toward more compact structural arrangements in both the Cid1-ATP and Cid1-UTP complexes.

**Figure 4:**
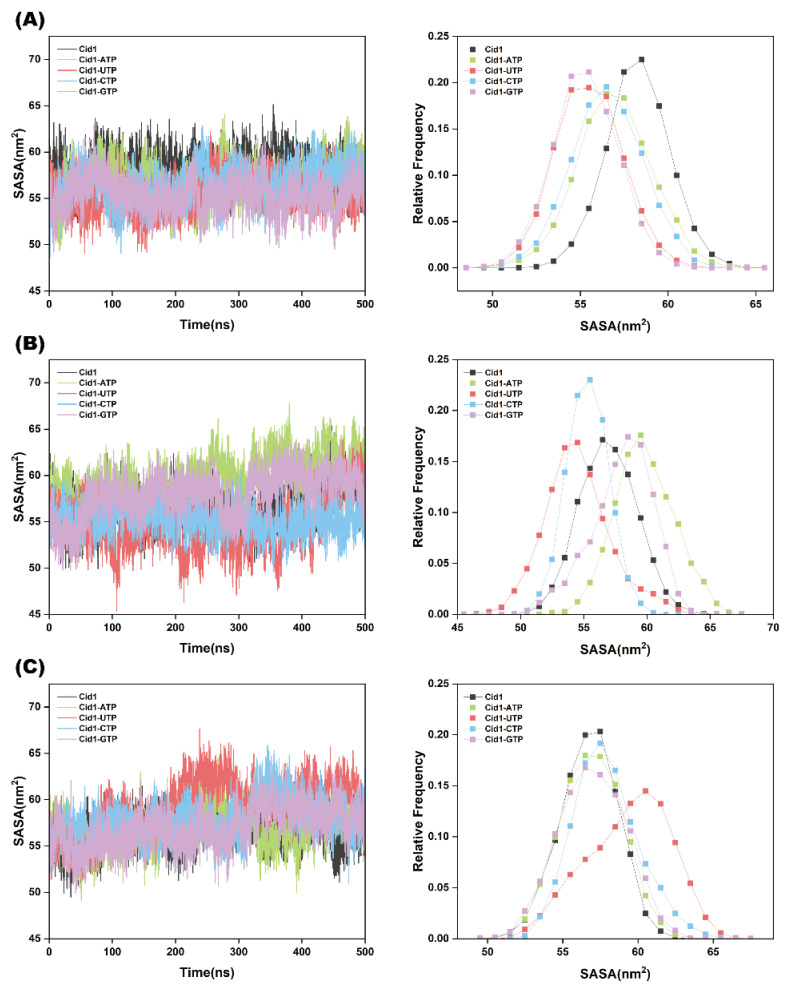
SASA plots of the five systems (Free-Cid1, Cid1-ATP, Cid1-UTP, Cid1-CTP, and Cid1-GTP) from three independent simulation replicates. (**A**) Replicate 1; (**B**) Replicate 2; (**C**) Replicate 3.

SASA is a surface area metric used to characterize the interactions between proteins and solvent molecules. When the SASA value is higher, it indicates a larger contact area between the protein and the solvent; conversely, a lower value signifies a smaller contact area. As shown in Figure 4A–C, the SASA differences among the four complexes—Cid1-ATP, Cid1-UTP, Cid1-CTP, and Cid1-GTP—were not significant. This phenomenon may be attributed to the distinct hand-shaped topology of the Cid1 enzyme, which results in a negligible difference in SASA between its open and closed states.

To ensure the reliability of the results and prevent accidental errors, we performed three independent simulations. We integrated the results of these three simulations and found that none of them exhibited significant fluctuations after 500 ns, indicating that the systems reached equilibrium beyond this time point. The results of the three simulations were plotted as RMSD and R_g_ graphs (Figure S2), which revealed no significant variations among the different simulation trajectories. Overall, after 500 ns of molecular dynamics simulations, all four systems demonstrated good stability and were suitable for further investigation.

To quantitatively assess the convergence and statistical reliability of these trajectories, comprehensive block averaging analysis [31,32,33] was performed on the complete 500 ns datasets (5000 frames at 0.1 ns/frame) for both RMSD and R_g_ metrics (Figure S3). The analysis employed block sizes ranging from 1 ns to 500 ns (10 to 5000 frames), demonstrating excellent convergence as evidenced by stable mean values across all block sizes. The standard errors plateaued at approximately 0.06 Å for RMSD, indicating well-estimated statistical uncertainties.

Overall, after 500 ns of molecular dynamics simulations, all systems demonstrated good stability and were suitable for further investigation.

### 2.4. NTP Structural Stability Analysis

**Figure 5:**
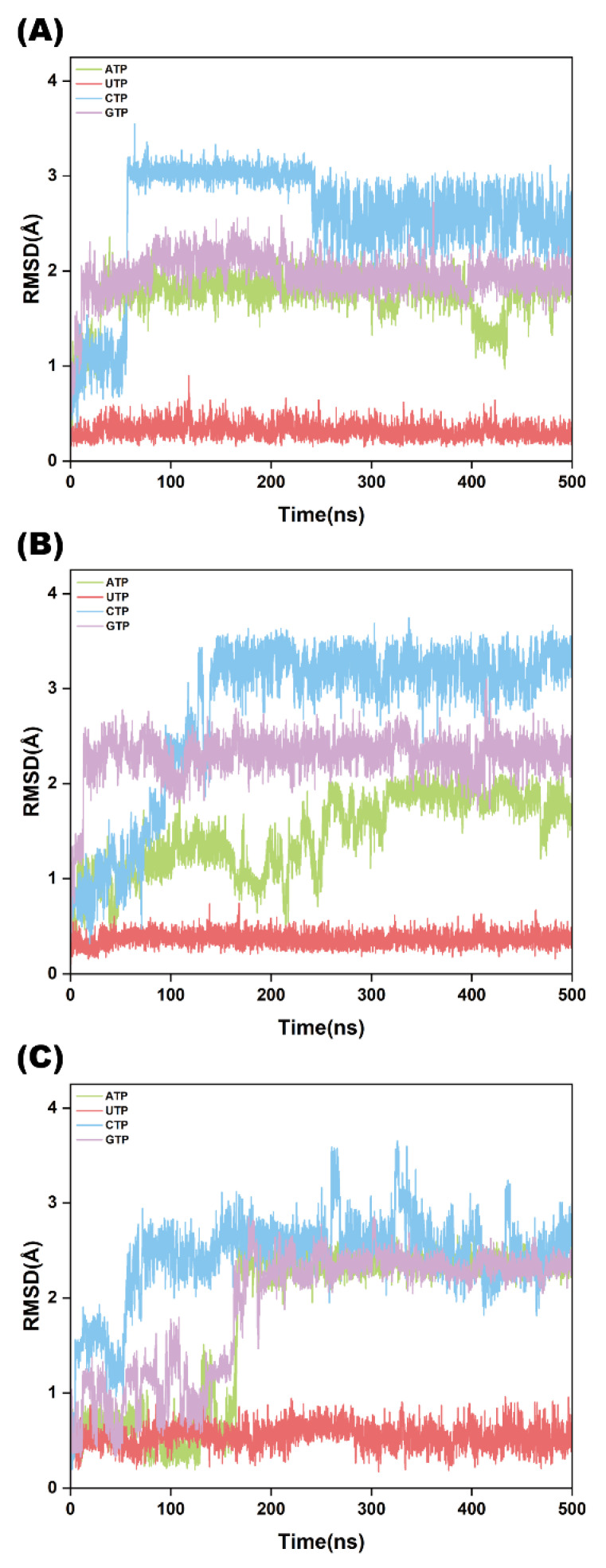
Ligand RMSD for the four NTP substrates across three independent simulation replicates. (**A**) Replicate 1; (**B**) Replicate 2; (**C**) Replicate 3.

The binding of NTP ligands renders the protein structure more compact and stable overall. Consequently, determining whether different substrates (ATP, UTP, CTP, GTP) exhibit comparable stability or significant differences is crucial for understanding substrate specificity. To address this, we investigated the stability of the four NTP ligands. As shown in Figure 5A, the RMSD values of ATP, UTP, CTP, and GTP predominantly fluctuated around 1.23 Å, 0.31 Å, 2.57 Å, and 2.00 Å, respectively. The data reveal that UTP displays the smallest fluctuation, followed by ATP, while CTP and GTP exhibit larger deviations. These results further confirm that Cid1-UTP exhibits the highest structural stability, followed by Cid1-ATP which maintains a relatively stable conformation among the NTP complexes. All replicates (Figure 5A–C) show consistent RMSD trends across the systems.

### 2.5. Binding Free Energy Calculation

This study employed the MM-PBSA method to evaluate the binding free energy of interactions between the four NTPs and the Cid1 protein. The results are presented in Table 1. It is important to note that the standard MM-PBSA approach does not calculate the entropic contribution (−TΔS), which typically leads to an overestimation of binding affinity (i.e., calculated ΔG values are more negative than experimental values). Therefore, the absolute free energy values reported here should be interpreted with caution and are used primarily for qualitative comparison of binding strengths between substrates [34,35].

Based on the equilibrated trajectories (450–500 ns), the binding free energies for the complexes formed between Cid1 and ATP, UTP, CTP, and GTP were −103.51 kcal/mol, −133.69 kcal/mol, −37.43 kcal/mol, and −68.84 kcal/mol, respectively. The results clearly indicate a more favorable (more negative) binding free energy for UTP compared to ATP, and for both UTP and ATP compared to CTP and GTP. This relative ranking likely reflects superior structural complementarity, enabling more stable substrate-enzyme interactions for UTP and ATP. ATP and UTP probably form optimized non-covalent interactions (e.g., hydrogen bonding, salt bridges) within Cid1’s catalytic pocket. Conversely, the higher binding free energies of CTP and GTP suggest weaker binding affinity, potentially due to less non-bonded interactions with the enzyme compared to ATP/UTP. Therefore, subsequent investigations will focus on analyzing specific non-covalent interactions to elucidate these differences.

The relative ranking of the MM-PBSA results further demonstrates that UTP and ATP are optimal substrates for Cid1, exhibiting more favorable binding affinity than CTP and GTP.

### 2.6. Distance Analysis

As a hand-shaped polymerase, Cid1 exhibits changes in the distance between its two structural domains when polymerizing different substrates. which can directly influence binding tightness between the substrate and the protein.

**Figure 6:**
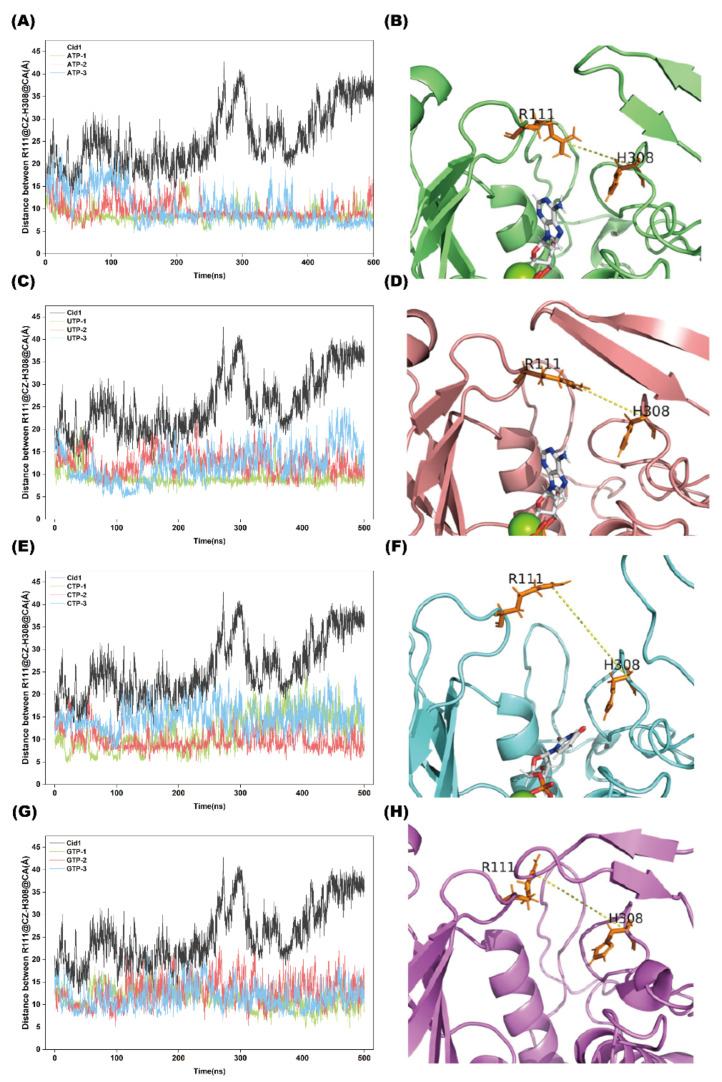
Distance analysis for the five systems across three independent simulation replicates. (**A**,**C**,**E**,**G**): Distances between R111@CZ and H308@CA for the Cid1-ATP, Cid1-UTP, Cid1-CTP, and Cid1-GTP systems with results from three replicates. (**B**,**D**,**F**,**H**): Representative secondary structure distances corresponding to each Cid1-NTP system.

As shown in Figure 6A–D, when ATP or UTP acts as the substrate, the distance between Cid1’s hand-shaped domains decreases, forming a more compact binding state. This structural contraction enhances substrate-enzyme binding stability, preventing dissociation of the substrate from the catalytic pocket.

In contrast, when CTP or GTP serves as the substrate (Figure 6E–H), the distance between domains of Cid1 maintains greater separation, resulting in an overall open conformation. This expanded structural state compromises binding stability. Under physiological conditions, substrates are more prone to dissociate from the enzyme’s active site.

### 2.7. Salt Bridge Analysis

As shown in Table 2, based on the geometric criteria defined in Materials and Methods, the salt bridge formation frequency in the Cid1-UTP and Cid1-ATP systems is significantly higher than that in the Cid1-GTP and Cid1-CTP systems. This clearly demonstrates that the differential substrate selectivity exhibited by the enzyme correlates with variations in non-covalent interactions across the different systems. Notably, salt-bridge occupancy was generally low (<3%) in all systems, indicating that such interactions occurred only infrequently during the simulations. This suggests that salt bridges may play a limited role in stabilizing the complexes, while the other non-covalent interactions previously mentioned (including hydrogen bonding, van der Waals, and electrostatic forces) are likely the dominant contributors to structural stability.

### 2.8. Hydrogen Bond Analysis

**Figure 7:**
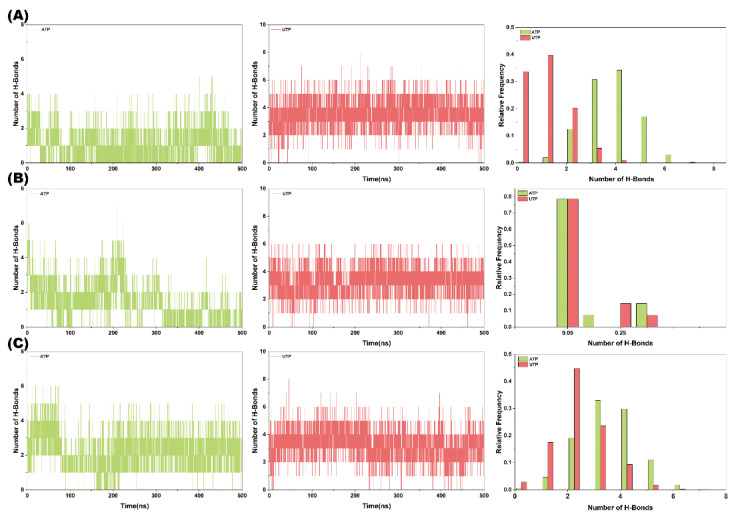
Hydrogen bond analysis for Cid1-ATP and Cid1-UTP systems across three independent replicates. (**A**) Replicate 1; (**B**) Replicate 2; (**C**) Replicate 3.

To elucidate the mechanism underlying the differential polymerization capabilities of the Cid1 protein towards ATP and UTP, this study conducted an in-depth investigation. As shown in Figure 7A–C, during the three replicated 500 ns GaMD simulations, the number of hydrogen bonds formed between Cid1 and UTP was significantly higher than that in the Cid1-ATP system. This observation suggests that UTP likely achieves enhanced binding stability with the enzyme through a greater number of hydrogen bond interactions. As a key form of non-covalent interaction, both the quantity and stability of hydrogen bonds directly influence the binding affinity and catalytic efficiency of protein-ligand complexes. Compared to ATP, UTP may form additional hydrogen bonds with residues in Cid1’s active site, enabling tighter binding and consequently increased substrate binding affinity.

### 2.9. Protein Residue Flexibility Analysis

**Figure 8:**
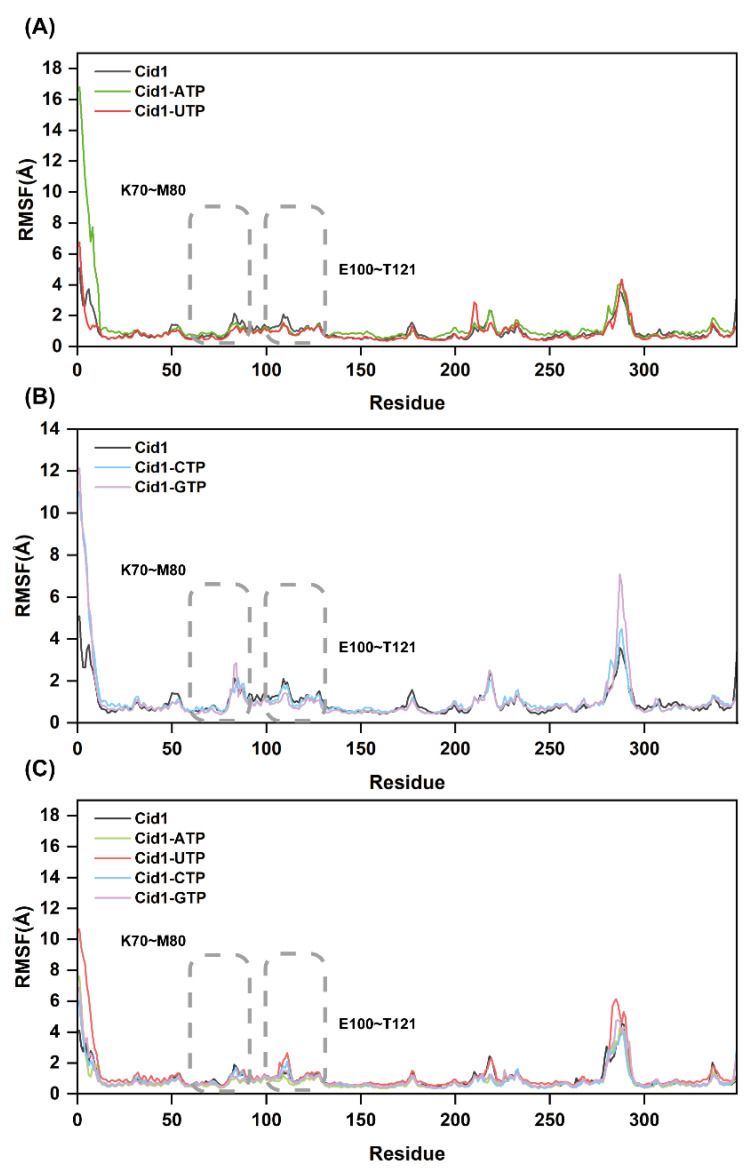
RMSF plots of the five systems (Free-Cid1, Cid1-ATP, Cid1-UTP, Cid1-CTP, and Cid1-GTP) from three independent simulation replicates. (**A**) Replicate 1; (**B**) Replicate 2; (**C**) Replicate 3.

Root Mean Square Fluctuation (RMSF) is a key metric for assessing protein structural flexibility. Generally, higher RMSF values indicate greater structural flexibility and more pronounced conformational changes, while lower RMSF values signify enhanced rigidity and conformational stability. To gain deeper insights into dynamic fluctuations of residues in Cid1 polymerase, we calculated RMSF values for all five systems using the equilibrated 50 ns trajectory (450–500 ns). As shown in Figure 8A,B, the free-Cid1 system exhibits globally higher RMSF values than the four ligand-bound systems. This observation indicates that free-Cid1 adopts greater structural and conformational flexibility, further confirming that NTP ligand binding enhances overall compactness and stability of the protein structure.

Furthermore, detailed analysis revealed significant atomic fluctuations in residues 70–80 and 100–121 across all five systems. These residues are positioned near Cid1’s catalytic pocket, suggesting that during substrate polymerization, the secondary structures surrounding the active site display high adaptability. Ligand binding not only restricts the conformational freedom of free-Cid1 but also enhances structural stability under functional conditions. The consistent RMSF profiles observed across all independent replicates (Figure 8A–C) further confirm the reproducibility of these dynamic structural features.

### 2.10. Secondary Structural Changes During Simulation

**Figure 9:**
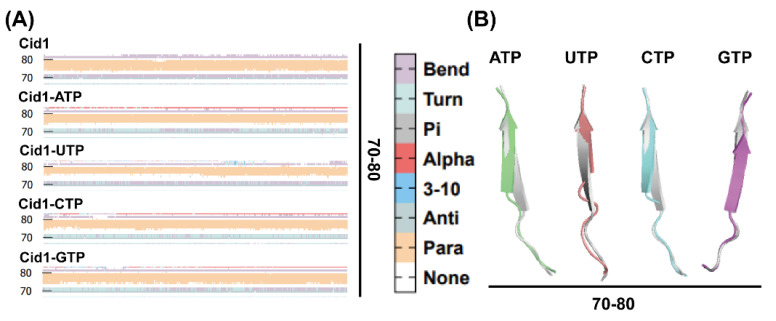
(**A**) DSSP changes in the five systems; (**B**) Secondary structure of the D70−E80 region, Free-Cid1 (silver gray), Cid1-ATP (green), Cid1-UTP (pink), Cid1-CTP (blue), Cid1-GTP (purple).

**Figure 10:**
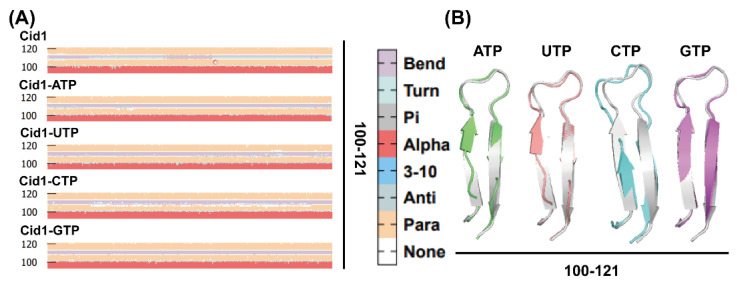
(**A**) DSSP changes in the five systems; (**B**) Secondary structure of the V100−K121 region, Free-Cid1 (silver gray), Cid1-ATP (green), Cid1-UTP (pink), Cid1-CTP (blue), Cid1-GTP (purple).

Building upon the RMSF analysis, we further employed Dictionary of Protein Secondary Structure (DSSP) to analyze secondary structural changes in residues 70–80 and 100–121. As shown in Figure 9 and Figure 10, upon binding with NTP substrates, both regions exhibited a universal shortening of β-sheets compared to the free-Cid1 system, albeit to varying degrees. Specifically, in the 70–80 region, all four NTP-bound complexes showed a reduction in β-sheet structure (Figure 9A,B), with the Cid1-UTP complex displaying the most pronounced effect, followed by Cid1-ATP and Cid1-CTP. A similar trend was observed in the 100–121 region, where the shortening of β-sheets was most evident in the Cid1-ATP and Cid1-UTP systems (Figure 10A,B), with Cid1-CTP exhibiting a moderate change. This universal phenomenon demonstrates that substrate binding induces local structural rearrangements across all complexes, optimizing the active site for interaction. The extent of this conformational adjustment, which likely accommodates the spatial and chemical requirements of the specific substrate, appears to correlate with binding affinity and may contribute to stabilizing the active site.

Secondary structure analysis not only reveals local conformations but also provides insights into functionally relevant active sites. Collectively, these results suggest that substrate-specific secondary structural changes may underlie enhanced catalytic activity when ATP is polymerized, which is a difference from conformational changes observed when other substrates are polymerized.

### 2.11. Analysis via NRIMD Web Server

**Figure 11:**
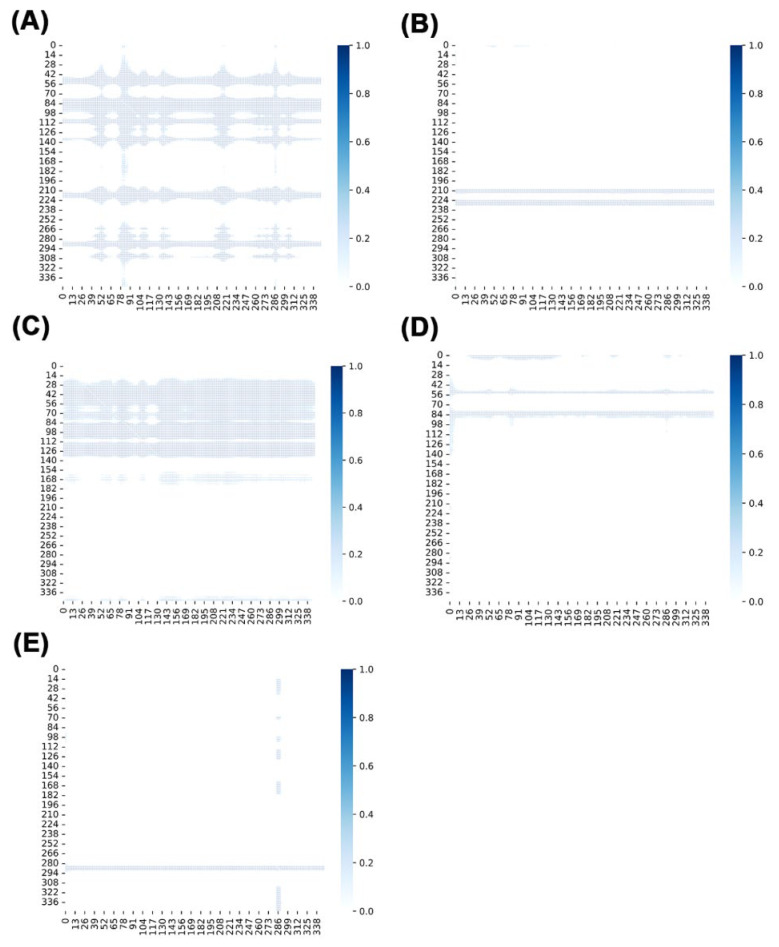
Plot of NRI modeling results for the five simulated systems; (**A**) Cid1; (**B**) Cid1-ATP; (**C**) Cid1-UTP; (**D**) Cid1-CTP; (**E**) Cid1-GTP.

The NRIMD web server implements an encoder-decoder architecture based on the NRI (Neural Relational Inference) model to analyze interaction distributions between protein residues using MD simulation trajectories. As shown in Figure 11A, the free-Cid1 system exhibits weak overall signals, indicating a loosely organized residue interaction network. Upon ATP binding (Figure 11B), significant signal enhancement occurs in specific regions such as residues 28–140, suggesting ATP binding to distinct sites induces variations in local functional networks. Conversely, UTP binding triggers (Figure 11C) pronounced signal amplification across multiple regions, reinforcing the global interaction network. This observation indicates UTP promotes holistic stabilization of the protein complex, confirming its role as primary substrate of Cid1. CTP binding elicits (Figure 11D) only localized enhancement such as residues 28–70, with limited spatial propagation, reflecting its restricted impact on protein regulation. GTP binding (Figure 11E) shows minimal signal changes compared to free-Cid1, demonstrating negligible influence on Cid1 proteins.

### 2.12. Quantum Chemical Calculations

**Figure 12:**
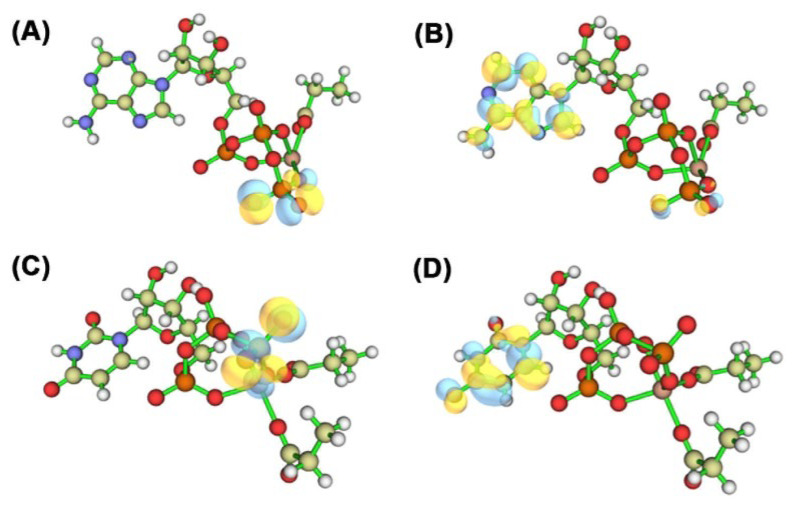
(**A**) Structure and HOMO orbitals of ATP; (**B**) Structure and LUMO orbitals of ATP; (**C**) Structure and HOMO orbital of UTP; (**D**) Structure and LUMO orbital of UTP.Yellow represents carbon atoms, blue represents nitrogen atoms, red represents oxygen atoms, brown represents phosphorus atoms, white represents hydrogen atoms, and the yellow and blue regions represent the positive and negative phases of the molecular orbital wave function, respectively.

Quantum Chemical Calculations constitutes a branch of computational chemistry that primarily employs principles of quantum mechanics to investigate the properties of molecules and chemical reactions [36]. To further explore the polymerization mechanism of Cid1 polymerase towards two NTPs (UTP and ATP), we conducted quantum chemical calculation analysis. The HOMO-LUMO energy gap refers to the energy difference between the HOMO and LUMO orbitals (Figure 12). The calculation results showed that the HOMO-LUMO energy gap of ATP (Figure 12A,B) was 40.69 kJ/mol, while that of UTP (Figure 12C,D) was 33.93 kJ/mol. A lower energy gap can reduce the barrier for electron transition, making electrons more prone to excitation and redistribution. Previous DFT studies have shown that smaller HOMO-LUMO gaps are generally associated with enhanced molecular reactivity and a greater tendency to form non-covalent interactions [37,38]. Thus, compared to ATP, UTP may be intrinsically more favorable for engaging in interactions such as hydrogen bonds and van der Waals forces. This provides a theoretical perspective that is consistent with the experimentally observed preference of Cid1 for UTP over ATP.

## 3. Materials and Methods

### 3.1. System Preparation

The sequence of Cid1 used in this study was obtained from the UniProt database (https://www.uniprot.org/, accessed on 15 April 2025) under sequence number O13833 [22,39]. AlphaFold 3 [30] was employed to predict the full-length protein structure and predict the binding structures of ATP, UTP, CTP, and GTP within the active site of Cid1. Subsequently, the structures of the Cid1-ATP, Cid1-UTP, Cid1-CTP, and Cid1-GTP complexes were generated by molecular docking using AutoDock Vina 1.2.0 [40]. The top-scoring pose from AutoDock Vina for each NTP was selected for subsequent simulations as it represents the most energetically favorable binding mode. Ultimately, five distinct systems were successfully constructed: System 1: Cid1 polymerase without ligand binding (Free-Cid1); System 2: Cid1 polymerase with bound ATP (Cid1-ATP); System 3: Cid1 polymerase with bound UTP (Cid1-UTP); System 4: Cid1 polymerase with bound CTP (Cid1-CTP); System 5: Cid1 polymerase with bound GTP (Cid1-GTP). In the latter four systems, the predicted structures included Mg^2+^ coordinated with oxygen atoms from both the nucleotide ligands and surrounding water molecules.

### 3.2. Molecular Docking

Molecular docking was performed using AutoDock Vina 1.2.0 to predict the binding modes of the four NTPs into the active site of the full-length Cid1 structure obtained from AlphaFold 3. Consequently, we obtained the complexes of Cid1-ATP, Cid1-UTP, Cid1-CTP, and Cid1-GTP.

### 3.3. Conventional Molecular Dynamics (cMD) Simulation

50 ns cMD simulations were performed on the five systems (Free-Cid1, Cid1-ATP, Cid1-UTP, Cid1-CTP, and Cid1-GTP) using the AMBER 22 software package [41]. During the simulation, the ff19SB force field [42] was applied to the protein, while the GAFF2 force field [43] was used for the four NTP ligands. The complexes were solvated using the TIP3P water model [44], and then placed in a cubic box with periodic boundary conditions, which can enable the simulation of a finite system while approximating the behavior of an infinite bulk environment [45,46,47,48,49]. To ensure simulation accuracy, an 8 Å buffer zone was specifically set between the complex and the box boundary. Ions were randomly added within the simulation box to achieve charge neutralization of the system. In order to eliminate possible atomic clashes in the initial structures, the particle mesh Ewald (PME) algorithm [50] was employed to efficiently handle long-range electrostatic interactions, with a cutoff radius set at 8 Å. Concurrently, the SHAKE algorithm [51] was applied to constrain hydrogen bonds, enhancing computational efficiency and maintaining system stability. MD simulations were performed with a time step of 2 fs [52], ensuring the precision of the simulation process. Analysis of the simulation trajectories was conducted using the CPPTRAJ module within the Amber package [53] combined with the VMD 1.9.3 [54] visualization tool. Each system underwent a 50 ns cMD simulation to sufficiently capture the dynamic behavior. This initial cMD simulation served two purposes: to equilibrate the systems and to collect the necessary statistics (mean and standard deviation of the potential energy) for calculating the GaMD acceleration parameters in the subsequent step [55].

### 3.4. Gaussian Accelerated Molecular Dynamics (GaMD) Simulation

After obtaining stable states for the Free-Cid1, Cid1-ATP, Cid1-UTP, Cid1-CTP, and Cid1-GTP systems through 50 ns cMD simulation, GaMD simulations were subsequently performed based on these equilibrated structures [55]. The protein was simulated using the ff19SB force field, the ligand molecules with the GAFF2 force field, and solvated in a TIP3P water box with an 8 Å buffer. Periodic boundary conditions were applied to all systems, and an appropriate number of counterions were added to achieve charge neutralization. A cutoff of 10 Å was set for non-bonded interactions to enhance simulation accuracy.

The production GaMD simulations were conducted in the NVT ensemble. This choice was motivated by the fact that the systems had already been fully equilibrated in terms of density and pressure during the preceding NPT cMD stage. Using NVT for production avoids introducing additional fluctuations from the barostat, which is crucial for obtaining a stable potential energy distribution—a prerequisite for robust reweighting of the GaMD simulations.

The key advantage of GaMD over cMD is its enhanced sampling efficiency. GaMD applies a harmonic boost potential to the system’s potential energy surface, effectively lowering energy barriers and accelerating conformational transitions. This allows for the observation of rare events, such as ligand binding and unbinding or protein large-scale conformational changes, within computationally feasible timescales that are often inaccessible to cMD.

Finally, each GaMD simulation was performed for a total duration of 500 ns, with atomic coordinates recorded every 100 ps to ensure sufficient data collection for subsequent analysis.

### 3.5. Binding Free Energy Calculation

The molecular mechanics/Poisson-Boltzmann surface area (MM-PBSA) method [56] was used to estimate ligand binding affinities. A total of 5000 frames were generated from the 500 ns trajectories, and snapshots from 4500 ps to 5000 ps were extracted every 10 frames (1000 frames in total) for analysis of protein-ligand complexes and individual components. The receptor was defined as residues 1–349, while the ligand was defined as residue 350–351. The Poisson-Boltzmann (PB) method was applied with an ionic strength of 0.15 M to approximate physiological conditions. Per-residue free energy decomposition was carried out (idecomp = 1), and the contributions of residues 1–351, including the ligand, were calculated (print res = 1–351) to analyze the detailed binding interactions. In these calculations, the binding free energy (ΔG_bind_) was calculated as:

ΔG_bind_ = ΔH − TΔS (1)

ΔH = ΔE_MM_ + ΔG_sol_ (2)

ΔE_MM_ = ΔE_ele_ + ΔE_vdW_ + ΔE_int_ (3)

ΔG_sol_ = ΔG_polar_ + ΔG_nonpolar_ (4)

Here, ΔE_MM_ represents gas-phase molecular mechanical energy, and ΔG_sol_ is the solvation free energy. The entropic term (TΔS) was not explicitly calculated, as normal mode or quasi-harmonic analyses are computationally demanding and often yield poorly converged results [34,39,57]. Instead, the relatively stable RMSD values and limited backbone fluctuations observed in our simulations suggest that conformational entropy changes are likely to be small, and thus the omission of the TΔS term is reasonable.

ΔE_MM_ includes covalent bond energy (ΔE_int_), electrostatic (ΔE_ele_), and van der Waals (ΔE_vdW_) contributions. Polar solvation energy (ΔG_polar_) was calculated using the PBSA module in AMBER 22 [58], while nonpolar solvation energy (ΔG_nonpolar_) was estimated via solvent-accessible surface area.

### 3.6. Trajectory Analysis

The Cpptraj [54,59] module in AMBER 22 was employed to perform trajectory analyses, including root mean square deviation (RMSD), root mean square fluctuation (RMSF), radius of gyration (R_g_), solvent-accessible surface area (SASA) and hydrogen Bond. The RMSF analysis was specifically conducted using the equilibrated portion of the trajectories (the last 50 ns) to ensure the assessment of residue flexibility was based on a stable conformational ensemble.

### 3.7. Salt Bridges Analysis

Salt bridges were identified using a geometrical definition. Specifically, a salt bridge was considered present when the distance between the nitrogen atom of a basic side chain (Lys/Arg) and the oxygen atom of an acidic side chain (Asp/Glu) was ≤3.2 Å, and the interaction angle was within 60–120°. The percentage values reported in Table 2 correspond to the fraction of MD snapshots that fulfilled these criteria. This geometrical cutoff has been widely applied in molecular dynamics studies to characterize salt-bridge formation [60,61,62].

### 3.8. NRIMD Web Server

To investigate the long-range regulatory mechanism from the binding site to the protein’s active center in the ligand binding process, we utilized the NRIMD web server (https://nrimd.luddy.indianapolis.iu.edu/, accessed on 16 June 2025) [63], a cloud-based platform for analyzing protein allosteric interactions based on MD simulations. This server employs a NRI (Neural Relational Inference) model [15] integrated with graph neural networks (GNNs) to process MD trajectories. The NRIMD web server converts the protein structure into a dynamic residue interaction graph and models the ligand binding process as a network of interacting residues. By minimizing the error between reconstructed trajectories and the original MD simulations, the server infers latent edges between residues, identifying key interactions and allosteric pathways. The learned embeddings efficiently capture the role of critical residues in conformational changes, providing robust insights into the ligand binding mechanism. For our analysis, we submitted the Cα atom trajectories from the complete MD simulations to the NRIMD web server. The platform’s deep learning framework enabled comprehensive analysis of long-range interactions, leveraging its user-friendly interface and high-performance backend to streamline the process. This approach significantly reduced the computational barrier while maintaining the accuracy and depth of the original methodology.

### 3.9. Quantum Chemical Calculations

Quantum chemical calculations for the four substrates were performed using Gaussian 09 [46] software. The method of calculation was in B3LYP state and the 6-31G* function. The HOMO-LUMO orbitals were visualized using the Multiwfn version 3.8 [47] tool. 

## 4. Conclusions

Combining 500 ns GaMD simulation with the NRIMD web server analysis and Quantum Chemical Calculations revealed that the significant differences in Cid1’s polymerization ability in the four substrates (ATP, UTP, CTP, and GTP) are mainly due to the following points:

First, the molecular docking results indicate that all four substrates form stable interactions with key residues such as SER183, SER62, and TYR184. When Cid1 polymerizes different substrates, UTP exhibits the highest number of interacting residues. This suggests that, under physiological conditions, UTP can bind tightly to the Cid1 protein and is less likely to dissociate from the catalytic pocket.

Second, through the analysis of protein structure stability and MM-PBSA analysis, it was found that the RMSD of Cid1-UTP is overall lower and more concentrated. At the same time, the distribution of R_g_ is more concentrated when UTP or ATP is the substrate, indicating that the enzyme structure is more compact at this time; the relative MM-PBSA binding free energy was most favorable for UTP, followed by ATP, and then GTP and CTP, which further indicates that UTP and ATP are more likely to stably bind with Cid1.

Third, hydrogen bonds further verified the energy advantage of UTP binding, especially UTP enabling Cid1 protein to reach a stable state more quickly.

Fourth, through NRIMD web server analysis, the binding of ATP and UTP to Cid1 significantly enhanced the signal of specific regions of the protein, and UTP also promoted the strengthening of the global network, indicating that it has an important influence on the protein polymerization. In contrast, CTP and GTP had relatively limited influence on the protein, showing smaller signal changes.

In conclusion, this paper reveals the substrate specificity of Cid1 for ATP, UTP, CTP, and GTP and its polymerization mechanism. UTP is the optimal substrate, ATP is the second-best substrate, and the superiority of its polymerization ability may stem from its affinity for the enzyme and catalytic efficiency, while other substrates show lower affinity and different catalytic characteristics.

## Figures and Tables

**Table 1:** Binding free energy MM-PBSA results (kcal/mol).

Systems	Cid1-ATP	Cid1-UTP	Cid1-CTP	Cid1-GTP
∆G_vdW_	−33.20 ± 0.53	−41.35 ± 0.64	−45.77 ± 0.59	−46.20 ± 0.57
∆G_ele_	123.74 ± 5.48	−186.07 ± 3.96	−319.88 ± 3.64	−2.92 ± 5.00
∆G_solv_	203.56 ± 5.14	728.83 ± 4.01	358.93 ± 3.30	406.24 ± 4.23
∆G_gas_	−307.07 ± 5.43	−862.52 ± 4.25	−396.36 ± 3.66	−475.08 ± 4.92
∆G_total_	−103.51 ± 0.85	−133.69 ± 0.78	−37.43 ± 0.73	−68.84 ± 1.44

**Table 2:** Salt bridge results for the four systems.

Systems	Saltbridge
Cid1-ATP	0.028
Cid1-UTP	0.028
Cid1-CTP	0.016
Cid1-GTP	0.024

## Data Availability

The original contributions presented in this study are included in the article and Supplementary Material. Further inquiries can be directed to the corresponding author(s).

## Supplementary Materials

The following supporting information can be downloaded at: https://www.mdpi.com/article/10.3390/ijms26199325/s1.
