# Supplementary material for: Gaussian Accelerated Molecular Dynamics Simulations Combined with NRIMD to Explore the Mechanism of Substrate Selectivity of Cid1 Polymerase for Different Nucleoside Triphosphates
Source: Int J Mol Sci. 2025 Sep 24;26(19):9325. doi: 10.3390/ijms26199325 (PMC12524604; doi:10.3390/ijms26199325)
Supplement: Supplementary file 1 [file ijms-26-09325-s001.zip › ijms-3838651-supplementary.pdf]

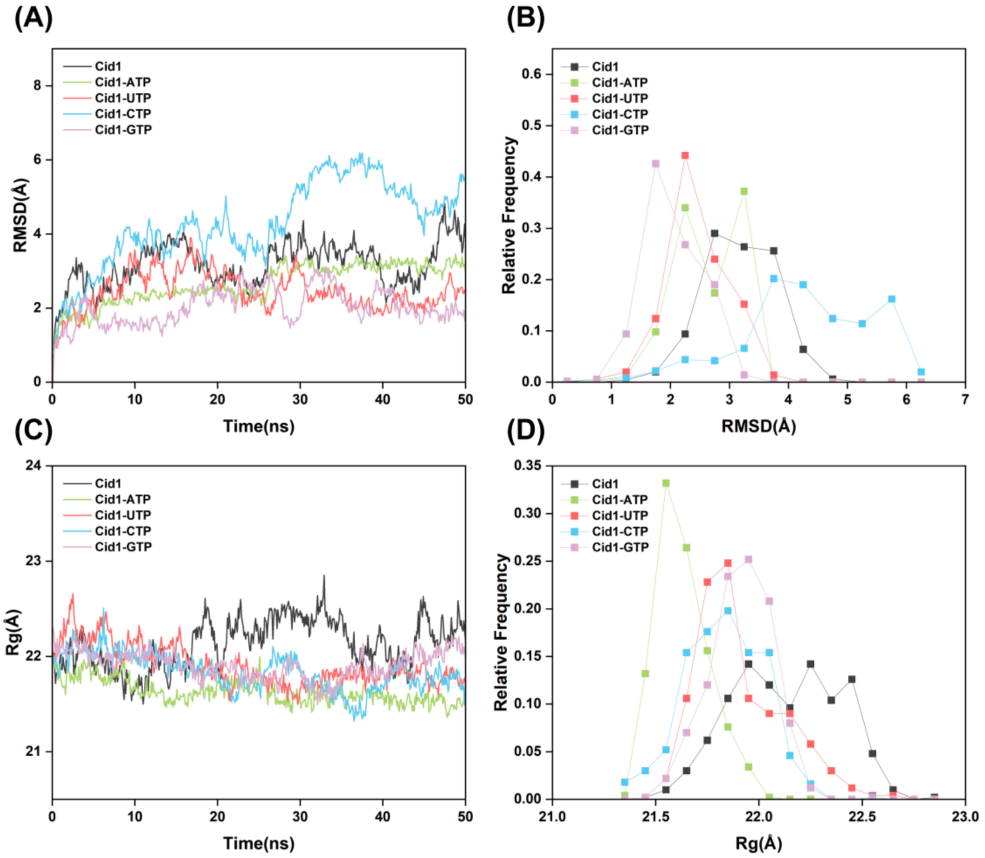

Figure S1 50 ns cMD simulation of the five simulated systems. (A) (B) RMSD plots for the five systems; (C) (D) R_g plots for the five systems.

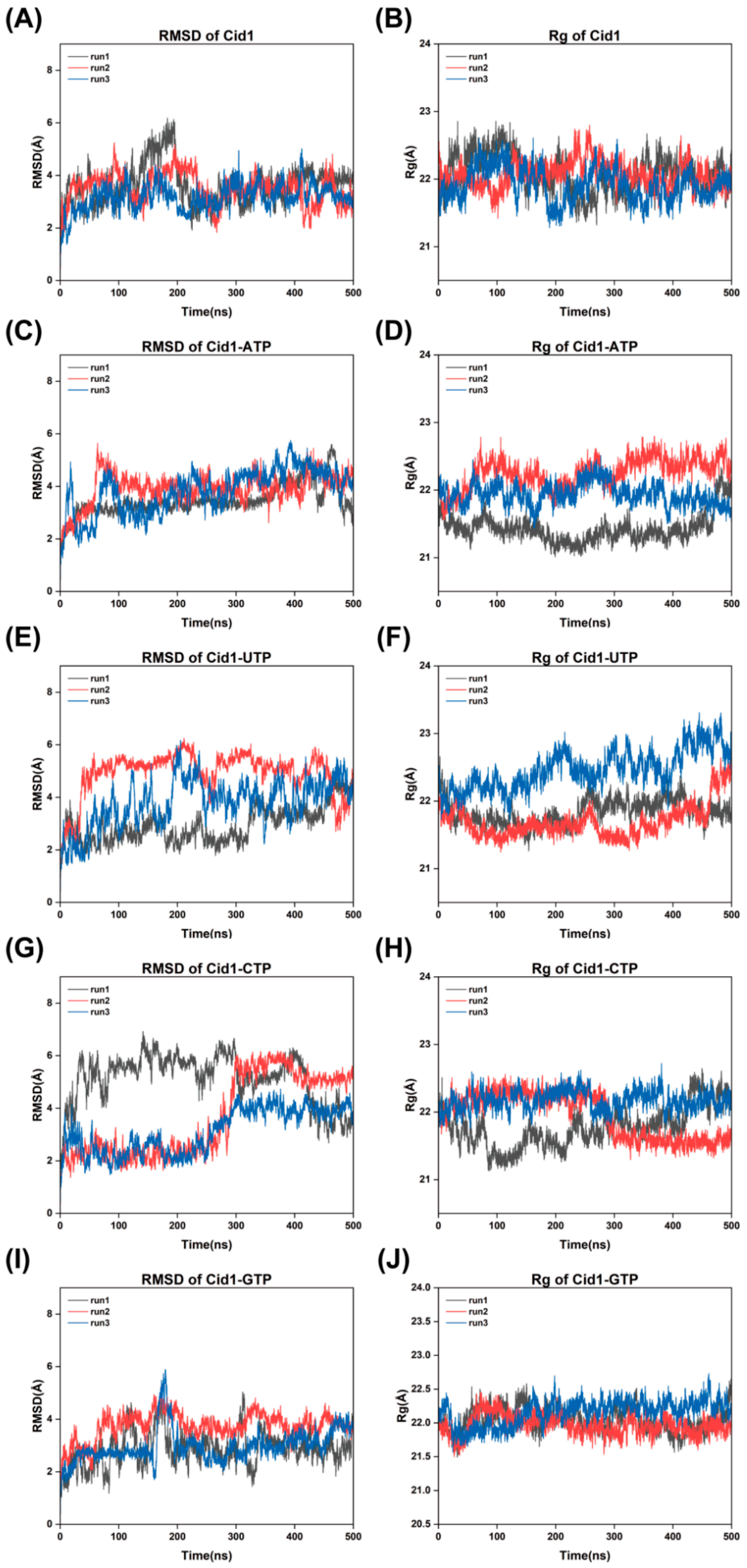

Figure S2 Results of three cMD and GaMD simulations plotted as RMSD and R_g graphs. (A) RMSD of Free-Cid1; (B) R_g of Free-Cid1; (C) RMSD of Cid1-ATP; (D) R_g of Cid1-ATP; (E) RMSD of Cid1-UTP; (F) R_g of Cid1-UTP; (G) RMSD of Cid1-CTP; (H) R_g of Cid1-CTP; (I) RMSD of Cid1-GTP; (J) R_g of Cid1-GTP;

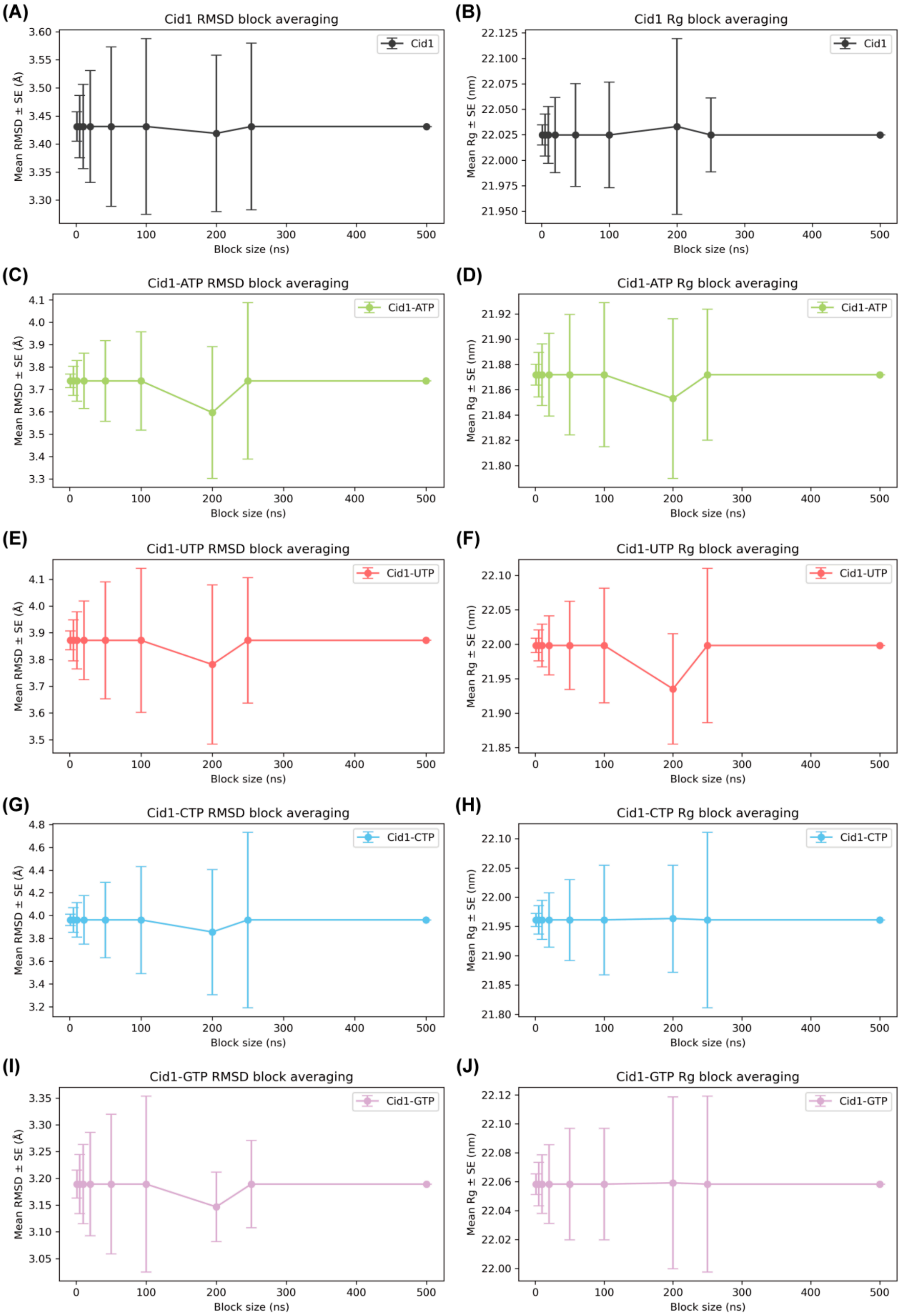

Figure S3 Results of block averaging analysis for RMSD and Rg values from three independent cMD and GaMD simulations. (A) RMSD of Free-Cid1; (B) R_g of Free-Cid1; (C) RMSD of Cid1-ATP; (D) R_g of Cid1-ATP; (E) RMSD of Cid1-UTP; (F) R_g of Cid1-UTP; (G) RMSD of Cid1-CTP; (H) R_g of Cid1-CTP; (I) RMSD of Cid1-GTP; (J) R_g of Cid1-GTP;
